# Symptoms in women with fibromyalgia after performing physical activity: the role of pain catastrophizing and disease impact

**DOI:** 10.1007/s10067-022-06342-5

**Published:** 2022-08-31

**Authors:** Irene López-Gómez, Lilian Velasco, Lorena Gutiérrez, Carmen Écija, Patricia Catalá, Cecilia Peñacoba

**Affiliations:** Department of Psychology, Rey Juan Carlos University, Avda. de Atenas s/n, 28922 Alcorcón, Madrid Spain

**Keywords:** Catastrophizing, Fatigue, Fibromyalgia, Pain, Walking

## Abstract

**Introduction:**

Walking is an effective treatment for symptoms’ management in patients with fibromyalgia. However, despite its benefits, fibromyalgia patients face a variety of obstacles that result in reduced ability to sustain physical exercise. The main goal of the study was to analyze the role of pain catastrophizing and fibromyalgia impact in the relationship between regular walking behavior and pain and fatigue experienced after a laboratory walking test.

**Method:**

The study has an observational analytical laboratory design. A total of 100 women were contacted by the research team. Seventy-six women diagnosed with fibromyalgia aged 18 years and older (mean age = 55.05, *SD* = 7.69) participated.

**Results:**

Significant correlations were found among regular walking behavior, pain catastrophizing, impact of fibromyalgia, pain intensity after walking, and fatigue intensity after walking. The serial multiple mediation analyses confirmed that pain catastrophizing and impact of fibromyalgia mediated the relationship between regular walking behavior and the level of pain (beta *B* = 0.044, 95% CI = [0.01–0.012]) and fatigue (beta *B* = 0.028, 95% CI = [0.01–0.08]) after the laboratory walking test. Also, the participants that walked less regularly experienced more pain and fatigue after the 6-Minute Walk Test.

**Conclusions:**

Considering cognitive variables alongside the impact of fibromyalgia will help understand the inhibitors of engaging in physical activity. Therapeutic walking programs must be tailored to patients with fibromyalgia to reduce pain and fatigue related to physical activity and to promote better functioning and quality of life.

**Table:** 

**Key Points** *• Regular walking behavior was associated with fibromyalgia impact, pain catastrophizing, and less pain and fatigue after physical activity*. *• When patients catastrophize pain, they usually interpret physical activity as threatening, which generates more pain and fatigue after doing exercise*. *• Therapeutic programs should be designed to reduce pain catastrophizing and fibromyalgia impact*.

**Supplementary Information:**

The online version contains supplementary material available at 10.1007/s10067-022-06342-5.

## Introduction

Fibromyalgia (FM) is a syndrome characterized by chronic widespread body pain, fatigue, sleeping problems, muscle weakness, and affective and cognitive dysfunction [1].

The absence of apparent pathological changes has led to the development of multi-component approaches recommended by different evidence-based guidelines for the management of the symptomatology [2]. These include physical exercise, along with pharmacological and psychological treatment [2]. It has been demonstrated that physical exercise is an effective treatment in pain management and stiffness, fatigue, and depression reduction in fibromyalgia patients [2]. More specifically, low- to moderate-intensity physical exercise such as walking has been shown to have positive effects on FM patients’ health status [3, 4].

Walking is a well-established aerobic activity for FM patients and a simple recommendation that promotes self-management as it is a self-regulated behavior [3]. It has also been shown that walking regularly reduces pain, fatigue, depression, and functional limitation [3–6]. Its benefits persist up to a year and it has shown even better results than other types of exercise in the follow-up periods [7].

Despite the benefits of walking, FM patients face a variety of symptoms that lead them to sedentary lifestyles, a low functional capacity, and a reduced ability to sustain physical exercise [8]. This highly disabling impact not only causes low rates of physical activity but also difficulties in performance [6–8]. Several factors that worsen performance have been reported. For example, Vlaeyen et al. [9] highlighted that fear of movement and (re)injury might reduce physical fitness due to activity avoidance which could lead the patient to no longer be able to perform physical activities because they could increase pain and suffering. Pain catastrophizing beliefs also stop physical activity; patients who catastrophize and interpret physical activity as threatening may show avoidance behavior and, thus, disability and reduced physical fitness, which in turn can increase pain and fatigue [10, 11]. In addition to the fear of pain, the new model of catastrophism proposed by Crombez et al. [12] also points out that the interference of this symptom in daily activities is a real impediment for patients to avoid physical exercise. Pain and fatigue have been considered as the most important inhibitors of walking behavior [11] and, although participants believed that walking could improve their health, they did not feel able to do it given their many physical impediments [13, 14].

To our knowledge, no studies have evaluated the influence of pain catastrophizing and FM impact perception in the pain or fatigue experienced after a walking test in a laboratory setting in women with FM [15]. Thus, consistent with past research [13, 15], the present study aimed to analyze the role of pain catastrophizing and FM impact in the relationship between regular walking behavior and pain and fatigue experienced after a laboratory walking test. We hypothesized that pain catastrophizing and FM impact will mediate the relationship between regular walking behavior and the level of pain and fatigue after walking. Following the fear-avoidance models [12, 15], we hypothesized that pain catastrophizing would be the first mediator as research has shown that it increases FM impact [10, 16, 17]. In turn, FM impact would be the second mediator of the model proposed. In sum, we expected those women who regularly walk more to experience less pain and fatigue after the laboratory test because they catastrophize less and have less FM impact.

## Materials and methods

### Study design

This study follows an observational analytical laboratory design.

### Participants

Participants were a total of 76 women diagnosed with FM with a mean age of 55.05 (age range = 32–69, *SD* = 7.69). Initially, 100 women diagnosed with FM were referred from different FM associations from the central area of Spain, and 23 of them did not participate in the study. The main reasons for not participating in the study were as follows: symptoms that prevented them from attending the day of the appointment (twelve women), lack of interest in the study (five women), and other unspecified reasons (six women). An additional subject was discarded due to its extreme scores. All participants had to meet the following criteria to be included in the study: (a) women, (b) with a diagnosis of fibromyalgia who fulfilled the 1990 or the 2010 American College of Rheumatology criteria for FM [1, 18, 19] (confirmed by a rheumatologist or a primary care physician), (c) over 18 years of age. Additionally, those women, who presented some type of physical comorbidity that prevented them from walking or whose walking behavior was contraindicated by their doctor, were excluded from the study. Furthermore, the existence of concomitant rheumatologic disorders, such as rheumatoid arthritis, systemic lupus erythematosus, Hashimoto’s disease, Sjogren’s syndrome, scleroderma, and reflex sympathetic dystrophy; and the existence of psychotic disorders, bipolar disorder, or any other serious psychiatric conditions were also part of the exclusion criteria of the study.

The sample size was calculated following Kline’s guideline [20] which indicated that 10 to 20 participants per estimated parameter would result in a sufficient sample. Additionally, the sample size for multiple regression was estimated using Daniel Soper’s sample size calculator [21]. According to the results, 35 subjects would be sufficient to detect an effect size of 0.4 assuming a 5% significance level and 85% power.

### Procedure

The study took place at the fibromyalgia unit of the university from January to April 2019 and the study’s protocol was approved by the ethics committee of this institution. The study was performed according to accepted guidelines on ethical practice and followed the Spanish Biomedical Research Act. Patients were informed of the study and, if they agreed to participate, they signed the informed consent and were then given an appointment at the fibromyalgia unit. During this appointment, a psychologist joined the participants to explain the assessment and answer their questions. First, a battery of pen-and-paper tests that took approximately 30 min to complete was administered. Afterwards, participants were asked to perform individually the 6-Minute Walk Test (6-MWT), and, lastly, they completed the pain and fatigue intensity scales. The 6-MWT is a clinically relevant measure of physical performance. This test has been commonly used in fibromyalgia patients [22, 23] and has been recommended by the Spanish Society of Rheumatology for Fibromyalgia [23, 24]. It measures the distance that each participant can walk on a flat surface in a period of 6 min. Patients are instructed to walk as fast as they can but comfortably for them. Therefore, they decide how fast they walk and are not induced to increase the speed. Every participant performed the 6-MWT individually and in the same time slot.

### Measures

Sociodemographic variables and clinical data were collected. Other variables were advocated using the following instruments.

#### Regular walking behavior

The International Physical Activity Questionnaire (IPAQ-S) was used to measure participants’ regular walking behavior in the last 7 days [25]. The one-item scale assessing daily time spent walking was used; consequently, no measures of internal consistency are provided.

#### Pain catastrophizing

The Spanish adaptation of the Pain Catastrophizing Scale (PCS) is a self-report instrument composed of 13 items on a 5-point Likert response format ranging from 0 (not at all) to 4 (always) [26]. This instrument is used to measure the amplification of painful sensations perceived from maladaptive cognitive, emotional, and behavioral processes. For this study, we used the total scale that showed an adequate internal validity with a Cronbach’s alpha of 0.93.

#### Impact of fibromyalgia

We used the Revised Fibromyalgia Impact Questionnaire (FIQ-R) that is a 21-item self-report instrument that represents the overall impact of symptoms on quality of life of women with FM [27]. The responses of the items are distributed in visual scales with 11 boxes whose discrete scores range from 0 to 10. For the present study, we used the total FIQ-R scale whose maximum score is 100 and which represents the global impact of fibromyalgia. A Cronbach’s alpha of 0.92 was found for the FIQ-R in this study.

#### Pain and fatigue intensity

The Brief Pain Inventory (BPI) was used to measure the intensity of perceived pain [28] and the Brief Fatigue Inventory (BFI) was used to measure the intensity of perceived fatigue. Both symptoms were evaluated after performing the 6-MWT. The intensity of pain and fatigue is recorded by an item in each questionnaire with a numerical scale that varies between 0 (“no pain or fatigue”) and 10 (“the greatest pain or fatigue imaginable”). Since these are one-item inventories, no measures of internal consistency are provided. The validity of the Brief Pain Inventory and Brief Fatigue Inventory has been revealed in previous research [28].

### Statistical analysis

First, descriptive analyses were calculated to determine the sociodemographic and clinical characteristics of the sample. Then, bivariate associations between the study variables (regular walking behavior, pain catastrophizing, impact of FM, pain and fatigue after 6-MWT) were explored with Pearson’s correlation analyses. Internal consistency analyses (Cronbach’s alpha coefficient) were performed for pain catastrophizing and impact of fibromyalgia. No missing data were found and one outlier was discarded due to its extreme scores. The Statistical package SPSS version 27 was used to perform these analyses. Next, simple mediation (model 4) was tested to determine the mediators between regular walking behavior and post level of pain or fatigue. In the following step, serial multiple mediation analysis (SMM) was used to explore causal chain linking mediators with a specific direction of causal flow (model 6). To perform these analyses, PROCESS macro version 3.4.1 was used with two significant mediators (pain catastrophizing and impact of FM).

According to Hayes [29], the bootstrap confidence intervals (CIs) estimates were based on 10,000 bootstraps to control for type I errors in the sample and a 95% CI was used. The proposed model is represented by the association between predictor (regular walking behavior) and mediator variables (mediator 1: pain catastrophizing (a_1_) and mediator 2: impact of FM (a_2_)); the association between mediators (pain catastrophizing is b_1_ and impact of FM is b_2_) and outcome variables (pain and fatigue after 6-MWT); “C” path represents the total effect of regular walking behavior on pain/fatigue after 6-MWT, having controlled for indirect effects (c) (effect of predictor on mediator 2 and effect of mediator 1 on outcome variables). Finally, “d” path represents the association between mediators (see figure of the proposed model in the Supplementary information).

## Results

### Descriptive characteristics of participants and correlations between study variables

Sociodemographic and clinical characteristics of participants are presented in Table 1. As can be seen, the majority profile corresponds to women with secondary studies (51.3%) and married (81.6%). The employment situation showed more variability, working at home (22.4%), employed (22.4%), and in sick leave (19.7%) being the most common profiles. In reference to clinical characteristics, participants had had an FM diagnosis for more than 12 years on average and they generally tended to take nonspecific medications related to glucose control, blood pressure, etc. (54.7%), along with other medications to relieve pain (24.3%) and anxious or depressive symptoms (21%).

**Table 1:** Sociodemographic and clinical characteristics (*n* = 76)

Sociodemographic variables	
Age, mean (*SD*)	55.05 (7.70)
Education level, *n* (%)	
No studies	4 (5.3%)
Primary	27 (35.5%)
Secondary	39 (51.3%)
University	6 (7.9%)
Marital status, *n* (%)	
Married	62 (81.6%)
Single	4 (5.3%)
Separated/divorced	7 (9.2%)
Widowed	3 (3.9%)
Employment status, *n* (%)	
Currently employed	17 (22.4%)
Unemployed	7 (9.2%)
Sick leave	15 (19.7%)
Retired	9 (11.8%)
Retired due to disability	11 (14.5%)
Housewife	17 (22.4%)
Clinical variables	
Time (years) since FM diagnosis, mean (*SD*)	12.32 (9.27)
Medication, *n* (%)	
Antidepressants/ anxiolytics	16 (21%)
Painkillers/anti-inflammatories	22 (24.3%)
Others (glucose, blood pressure, antihistamines…)	38 (54.7%)

*FM*, fibromyalgia

Correlation analyses showed relationships between regular walking behavior, pain catastrophizing, impact of FM, and pain and fatigue after 6-MWT. Specifically, regular walking behavior was negatively associated with pain (*p* = 0.05), fatigue (*p* = 0.01), pain catastrophizing (*p* = 0.01), and impact of FM (*p* = 0.01). Pain was positively associated with fatigue (*p* = 0.01), impact of FM (*p* = 0.01), and pain catastrophizing (*p* = 0.01). In the same way, fatigue was positively associated with impact of FM (*p* = 0.01) and pain catastrophizing (*p* = 0.05). Finally, pain catastrophizing was positively associated with impact of FM (*p* = 0.05). As recommended by Baron and Kenny [30], the chosen mediators significantly correlated with the predictors and the outcome variables.

### Simple mediation and serial multiple mediation analysis

First, the mediating role of pain catastrophizing in the relationship between regular walking behavior and pain/fatigue after 6-MWT was examined. The total and direct effect of regular walking behavior on pain (*t* =  − 2.21, *p* = 0.03), the effect of regular walking behavior on pain catastrophizing (*t* =  − 1.96, *p* = 0.045), and the effect of pain catastrophizing on pain (*t* = 2.51, *p* = 0.01) were significant. With fatigue as an outcome variable, a significant total and direct effect were found (*t* =  − 2.50, *p* = 0.01); moreover, the effect of regular walking behavior on pain catastrophizing (*t* =  − 1.96, *p* = 0.043) and the effect of this mediator on fatigue (*t* = 1.50, *p* = 0.015) were also significant.

Second, impact of FM was also examined as a mediator between regular walking behavior and pain/fatigue after 6-MWT. The total and direct effect (*t* =  − 2.21, *p* = 0.03), the effect of regular walking behavior on FM impact (*t* =  − 2.38, *p* = 0.02), and the effect of this mediator on pain (*t* = 7.97, *p* = 0.000) were significant. The same occurs with fatigue as an outcome variable; the total and direct effect was significant (*t* =  − 1.98, *p* = 0.045); furthermore, the effect of regular walking behavior on FM impact (*t* =  − 2.33, *p* = 0.022) and the effect of this mediator on fatigue (*t* = 4.13, *p* = 0.001) were also significant.

As can be seen in Table 2, regular walking behavior had a significant indirect effect on pain/fatigue after 6-MWT, with pain catastrophizing and impact of FM as mediators.

**Table 2:** Simple mediation effects of regular walking behavior on pain or fatigue after 6-Minute Walk Test

Bootstrapping					
	Point estimated	BC 95% CI		BCa 95% CI	
		Lower	Upper	Lower	Upper
Pain					
Total indirect effects					
Pain catastrophizing	0.039	0.001	0.008	0.014	0.150
Impact of FM	0.042	0.018	0.023	0.308	0.334
Fatigue					
Total indirect effects					
Pain catastrophizing	0.032	0.006	0.009	0.107	0.160
Impact of FM	0.050	0.012	0.020	0.170	0.215

The point estimate is the indirect effect calculated in the original sample; *CI*, confidence interval; *BC*, bias corrected; *BCa*, bias corrected and accelerated; *FM*, fibromyalgia

**Fig. 1:**
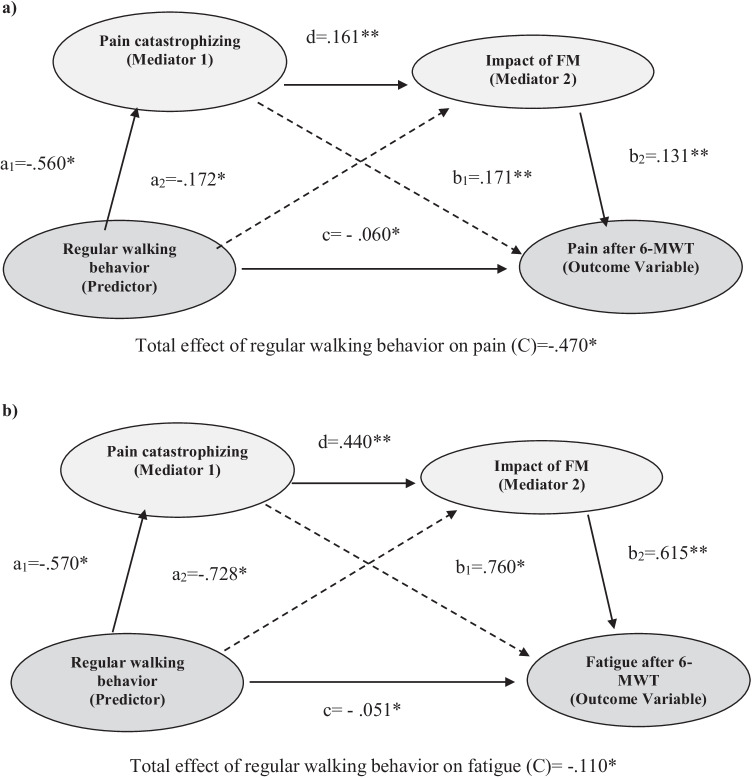
Path diagram illustrating SMM1 (pain) and SMM2 (fatigue). Note: *p < 0.05; ** *p* < 0.01. a_1_a_2_, relation between predictor (walking behavior) on mediator 1 (pain catastrophizing) and mediator 2 (impact of FM); b_1_b_2_, relation between mediator 1 and mediator 2 on outcomes (pain and fatigue); c, indirect effect of mediators on outcomes; d, relation between mediators; C, total effect of predictor on outcomes with mediators; FM, fibromyalgia; SMM, serial multiple mediation analysis; 6-MWT, 6-Minute Walk Test

Based on previous literature [16, 17], we propose two serial multiple mediation models (SMM) defined by pain catastrophizing (M_1_) and impact of FM (M_2_) on pain (SMM_1_) and fatigue after 6-MWT as outcome variables (SMM_2_). All indirect effects were significant in both models for regular walking behavior on pain/fatigue. However, when we compared the contrast effects of SMM_1_ and SMM_2_, it suggested that the indirect effect 3 (predictor→M_1_→M_2_→outcome variable) was stronger than the indirect effect of simple mediation models with pain (*B* = 0.044, 95% CI = 0.010–0.012) and fatigue (*B* = 0.028, 95% CI = 0.010–0.080). This means that SMM_1_ and SMM_2_ showed a stronger mediating effect of pain catastrophizing when we include the effect of impact of FM in comparison to the effect of each mediator separately (see Fig. 1).

## Discussion

This study aimed to analyze the role of pain catastrophizing and FM impact on the relationship between regular walking behavior and pain and fatigue experienced after a laboratory walking test. Significant associations were found between all the study variables supporting our hypothesis. In line with previous research [2, 5], regular walking behavior was associated with less pain and fatigue after physical activity, FM impact, and pain catastrophizing. The correlation between pain and fatigue was the strongest correlation found, showing that these symptoms are closely linked, and both are essential in the understanding of FM [18].

Furthermore, the study hypothesis stated that pain catastrophizing and FM impact will mediate the relationship between regular walking behavior and the level of pain and fatigue after the laboratory walking test. The serial multiple mediation (SMM) analyses performed confirmed this hypothesis. Following the fear-avoidance models [12, 15], our results pointed out that pain catastrophizing beliefs stop patients from engaging in physical activity, and this increases FM impact [9, 16, 17]. Previous research has also shown that pain catastrophizing is related to higher levels of pain and fatigue [13].

To our knowledge, no research has explored a very important and unanswered question: Are the pain and fatigue suffered after physical activity influenced by pain catastrophizing and FM impact? According to our results, the answer seems to be affirmative. When patients catastrophized pain, they usually interpreted physical activity as threatening which generated more pain and fatigue immediately after doing exercise. Additionally, the present study considered the effect of regular walking behavior, stating that the participants that walked less regularly suffered more pain and fatigue after 6-MWT. The association between the distance walked in the 6-MWT and pain, FM impact, and physical impairment has been supported by several studies, but no information was provided regarding the pain and fatigue after doing the test [22, 23]. These two symptoms have been considered to be the most important inhibitors of walking in FM patients [8, 14], and since the subjects do not walk to avoid pain and fatigue, it results in a reduced practice of walking and poor adherence to it [8]. Unfortunately, when patients do not walk regularly, they confirm their fears, suffering from pain and fatigue after walking [13, 14]. The present study showed how regular walking is associated with less pain and fatigue after a walking test, offering evidence on how to break the vicious cycle FM patients usually find themselves in.

One of the strengths of this study was the use of an objective measure of functional capacity, the 6-MWT. Moreover, this test has been commonly used in fibromyalgia patients [23, 24] and has been recommended by the Spanish Society of Rheumatology for Fibromyalgia [24]. This measure is ecologically valid as well as clinically feasible because it requires minimal instrumentation and time. As in previous studies, the study was designed to test the relationships in FM patients between cognitions, behaviors, and symptomatology to improve interventions and help patients live with more satisfaction.

### Study limitations

Concerning the limitations of the study, the nature of the study design (i.e., observational analytical laboratory study) helps establish the possible direction of associations, but results should be confirmed in future longitudinal studies. The fact that the sample was only composed of women and that it was a convenience sample could limit the generalizability of the results. However, most FM patients are women, and the sample was contacted through several patients’ associations that are very common among FM patients [31]. Previous research has shown that the characteristics of patients from associations should be considered equivalent to the ones of people with FM in the general population [6]. Additionally, the sample size was relatively small but similar to the sample sizes of previous studies that have used the 6-MWT in patients with chronic pain [32].

### Conclusions

In conclusion, the study results showed that pain catastrophizing and FM impact were mediators in the relationship between regular walking behavior and the level of pain and fatigue after a laboratory walking test in this sample. Therefore, the present study provided evidence in favor of considering cognitive variables and the impact of the syndrome in patients’ quality of life to better understand the common FM patients’ complaint of not walking because of the pain and fatigue felt afterwards. The main goal of this research was to untangle this relationship to clarify the path of designing interventions that could help patients overcome these obstacles and improve their quality of life.

## Supplementary Information

Below is the link to the electronic supplementary material.

Supplementary file1 (DOC 70 KB)

## References

[1] Wolfe F, Clauw DJ, Fitzcharles MA (2010). The American College of Rheumatology preliminary diagnostic criteria for fibromyalgia and measurement of symptom severity. Arthritis Care Res.

[2] Häuser W, Thieme K, Turk DC (2010). Guidelines on the management of fibromyalgia syndrome - a systematic review. Eur J Pain.

[3] Peñacoba C, Pastor-Mira MA, López-Roig S, Sanz Y, Velasco L (2019). Healthcare provider advice to engage in walking regimens and adherence in women with fibromyalgia. Rehab Nursing.

[4] Santos E, Campos MA, Párraga-Montilla JA, Aragón-Vela J, Latorre-Román PA (2020). Effects of a functional training program in patients with fibromyalgia: a 9-year prospective longitudinal cohort study. Scand J Med Sci Sports.

[5] Busch AJ, Webber SC, Brachaniec M (2011). Exercise therapy for fibromyalgia. Curr Pain Headache Rep.

[6] Pastor MA, López-Roig S, Sanz Y (2015). Andar como forma de ejercicio físico en la Fibromialgia: un estudio de identificación de creencias desde la Teoría de la Acción Planeada. [Walking as physical exercise in fibromyalgia: an elicitation study from the theory of planned behavior]. Anal Psicología.

[7] Kayo AH, Peccin MS, Sanches CM, Trevisani VF (2012). Effectiveness of physical activity in reducing pain in patients with fibromyalgia: a blinded randomized clinical trial. Rheumatol Int.

[8] López-Roig S, Pastor MÁ, Peñacoba C, Lledó A, Sanz Y, Velasco L (2016). Prevalence and predictors of unsupervised walking and physical activity in a community population of women with fibromyalgia. Rheumatol Int.

[9] Vlaeyen JWS, Kole-Snijders AMJ, Boeren RGB, van Eek H (1995). Fear of movement/(re)injury in chronic low back pain and its relation to behavioral performance. Pain.

[10] Esteve R, López-Martínez AE, Peters ML (2017). Activity pattern profiles: relationship with affect, daily functioning, impairment, and variables related to life goals. J Pain.

[11] Sanz-Baños Y, Pastor MÁ, Velasco L (2016). To walk or not to walk: insights from a qualitative description study with women suffering from fibromyalgia. Rheumatol Int.

[12] Crombez G, De Paepe AL, Veirman E, Eccleston C, Verleysen G, Van Ryckeghem DML (2020). Let’s talk about pain catastrophizing measures: an item content analysis. PeerJ.

[13] Häuser W, Klose P, Langhorst J (2010). Efficacy of different types of aerobic exercise in fibromyalgia syndrome: a systematic review and meta-analysis of randomised controlled trials. Arthritis Res Ther.

[14] Pastor-Mira MA, López-Roig S, Peñacoba C, Sanz-Baños Y, Lledó A, Velasco L (2020). Predicting walking as exercise in women with fibromyalgia from the perspective of the theory of planned behavior. Women Health.

[15] Crombez G, Eccleston C, Van Damme S, Vlaeyen JW, Karoly P (2012). Fear-avoidance model of chronic pain: the next generation. Clin J Pain.

[16] Écija C, Luque-Reca O, Suso-Ribera C, Catala P, Peñacoba C (2020). Associations of cognitive fusion and pain catastrophizing with fibromyalgia impact through fatigue, pain severity, and depression: an exploratory study using structural equation modeling. J Clin Med.

[17] Velasco L, López-Gómez I, Gutiérrez L, Écija C, Catalá P, Peñacoba C (2021). Exploring the preference for fatigue-avoidance goals as a mediator between pain catastrophizing, functional impairment, and walking behavior in women with fibromyalgia. Clin J Pain.

[18] Wolfe F, Smythe HA, Yunus MB (1990). The American College of Rheumatology 1990 criteria for the classification of fibromyalgia. Report of the Multicenter Criteria Committee. Arthritis Rheum.

[19] Wolfe F, Walitt BT, Katz RS, Häuser W (2014). Symptoms, the nature of fibromyalgia, and diagnostic and statistical manual 5 (DSM-5) defined mental illness in patients with rheumatoid arthritis and fibromyalgia. PLoS ONE.

[20] Kline RB (1998). Principles and practice of structural equation modeling.

[21] Soper DS (2022) A-priori sample size calculator for multiple regression [software] 2022. Available from: https://www.danielsoper.com/statcalc. Accessed 19 July 2022

[22] Mannerkorpi K, Svantesson U, Broberg C (2006). Relationships between performance-based tests and patients’ ratings of activity limitations, self-efficacy, and pain in fibromyalgia. Arch Phys Med Rehabil.

[23] Pankoff B, Overend T, Lucy D, White K (2000). Validity and responsiveness of the 6 minute walk test for people with fibromyalgia. J Rheumatol.

[24] Rivera J, Alegre C, Ballina FJ (2006). Documento de consenso de la Sociedad Española de Reumatología sobre la fibromialgia. Reumatol Clin.

[25] Craig CL, Marshall AL, Sjöström M (2003). International physical activity questionnaire: 12-country reliability and validity. Med Sci Sports Exerc.

[26] García Campayo J, Rodero B, Alda M (2008). Validación de la versión española de la escala de la catastrofización ante el dolor (Pain Catastrophizing Scale) en la fibromialgia [Validation of the Spanish version of the Pain Catastrophizing Scale in fibromyalgia]. Med Clin.

[27] Salgueiro M, García-Leiva JM, Ballesteros J, Hidalgo J, Molina R, Calandre EP (2013). Validation of a Spanish version of the Revised Fibromyalgia Impact Questionnaire (FIQR). Health Qual Life Outcomes.

[28] Cleeland CS, Ryan KM (1994). Pain assessment: global use of the Brief Pain Inventory. Ann Acad Med Singap.

[29] Hayes AF (2013). Introduction to mediation, moderation, and conditional process analysis: a regression-based approach.

[30] Baron RM, Kenny DA (1986). The moderator-mediator variable distinction in social psychological research: conceptual, strategic, and statistical considerations. J Person Social Psychol.

[31] Rivera Redondo J (2012) La fibromialgia en el sistema sanitario español. Generalidades e impacto en la calidad de vida. In: Peñacoba C (ed) Fibromialgia y promoción de la salud: Herramientas de intervención psicosocial. Dykinson, Madrid, Spain, pp 45–73

[32] McCormick ZL, Gagnon CM, Caldwell M (2015). Short-term functional, emotional, and pain outcomes of patients with complex regional pain syndrome treated in a comprehensive interdisciplinary pain management program. Pain Med.

