# Supplementary material for: Symptoms in women with fibromyalgia after performing physical activity: the role of pain catastrophizing and disease impact
Source: Clin Rheumatol. 2022 Aug 31;42(1):225–32. doi: 10.1007/s10067-022-06342-5 (PMC9823036; doi:10.1007/s10067-022-06342-5)
Supplement: Supplementary file 1 — Supplementary file1 (DOC 70 KB) [file 10067_2022_6342_MOESM1_ESM.doc]

**Supplementary information**

Proposed model for regular walking behavior-pain/fatigue according to pain catastrophizing and impact of FM as mediators

*d_21_*

Pain Catastrophizing

Impact of FM

Pain/Fatigue

*a_1_*

*a_2_*

*b_1_*

*b_2_*

*c’*

Regular walking behavior

*Note: FM* fibromyalgia.
